# Visualizing large choroidal blood flow by subtraction of the choriocapillaris projection artifacts in swept source optical coherence tomography angiography in normal eyes

**DOI:** 10.1038/s41598-018-34102-6

**Published:** 2018-10-24

**Authors:** Ichiro Maruko, Taizo Kawano, Hisaya Arakawa, Taiji Hasegawa, Tomohiro Iida

**Affiliations:** Department of Ophthalmology, Tokyo Women’s Medical University School of Medicine, 8-1 Kawada-cho, Shinjuku, Tokyo, Japan

## Abstract

Optical coherence tomography angiography (OCTA) seems not to image the choroidal blood flow pattern in the normal individual because of the OCT light attenuation. Our purpose in the current study was to visualize the large choroidal blood flow pattern after subtraction of the choriocapillaris projection artifact in normal eyes non-invasively by swept source (SS) OCTA. Sixty-one eyes of 45 individuals (19 men, 26 women) without ocular disease were examined by SS-OCTA (AngioPlex Elite 9000, Zeiss, Germany). A 12 × 12 mm macular area was scanned. Subfoveal choroidal thickness (SCT) was measured, and the choroidal blood flow pattern in a slab of 30 µm width at one-half of SCT was analyzed. In examining the choroidal blood flow pattern, a slab that was between 30 to 60 µm posterior to the retinal pigment epithelium, in which the choriocapillaris blood flow was most clearly imaged, was used for the subtraction of the projection artifacts from the choriocapillaris on the stromal area of choroid. The ratio (%) of the choroidal blood flow area in the whole choroidal region was calculated after binarization. Thirty-four eyes of 27 individuals (12 men, 15 women) were also examined by spectral domain OCTA (SD-OCTA). After the subtraction, the middle and large choroidal blood flow were clearly visible in SS-OCTA in all eyes. The mean SCT was 297 ± 61 µm, and the mean ratio of the choroidal blood flow area was 27.3 ± 8.2%, which was significantly correlated with SCT (R = 0.738, *P* < 0.01). SD-OCTA did not show the choroidal blood flow pattern. In conclusion, removal of the projection artifacts of choriocapillaris can make the choroidal blood flow visible in SS-OCTA of normal eyes. Because the ratio of choroidal blood flow area was correlated with SCT, the choroidal blood flow might be an important factor related to the choroidal thickness.

## Introduction

Optical coherence tomography angiographic (OCTA) instruments have become commercially available, and they can be used to obtain images of the vascular patterns of the retina and choroid by evaluating the changes in successive OCT images^1,2^. The reflections from stationary tissue do not change over time while the blood flow causes changes in the degree of reflection over time. Large changes in flow produce correspondingly large changes in the reflection from one image to the next, and lower flow rates produce correspondingly smaller changes in the reflection. The movements of the patient caused by breathing and cardiac pulsations have been compensated by effective eye tracking software. Signal attenuation, either by light absorption from the ocular pigments or loss of coherence from multiple scattering, can reduce the absolute magnitude of the changes being measured. The flow signal is believed not to be attainable from the larger choroidal vessels without defects in the retinal pigment epithelium (RPE)^3–8^. At this moment, the blood flow patterns of the large choroidal vessels have not been obtained from emmetropic eyes by the commercially available OCTA devices. Instead, now standard en face OCT images might be used to evaluate the choroidal vascular structure itself without blood flow information.

Although the large or medium choroidal blood vessels appear as black structures in the standard OCTA images of the segmented intermediate choroidal slab, it is possible to image the choroidal vascular pattern as weak white patches especially in the swept source (SS) OCT devices using long wavelength observations lights. We have questioned for some time why the tissue surrounding the choroidal vessels appeared to be white in the OCTA images segmented for the intermediate choroidal area. These are projection artifacts that are formed by the alterations in the light intensity being reflected back by some deeper tissue than the actual vascular structure. The choriocapillaris is the only tissue with uniform and fine blood flow anterior to the choroidal vessel. We suggest that these whitish images surrounding the choroidal vessels were due to projection artifacts of the blood flow in the choriocapillaris to the stromal tissue of the choroid. Thus, we reasoned that if these projection artifacts could be removed from the images, a better image of the large choroidal vessels would be obtained.

Thus, the purpose of this study was to determine whether the subtraction of the choriocapillaris projection artifacts will make the deeper large choroidal vessels more visible. To accomplish this, we evaluated the SS-OCTA images after the subtraction of the choriocapillaris projected artifacts.

## Results

**Figure 1:**
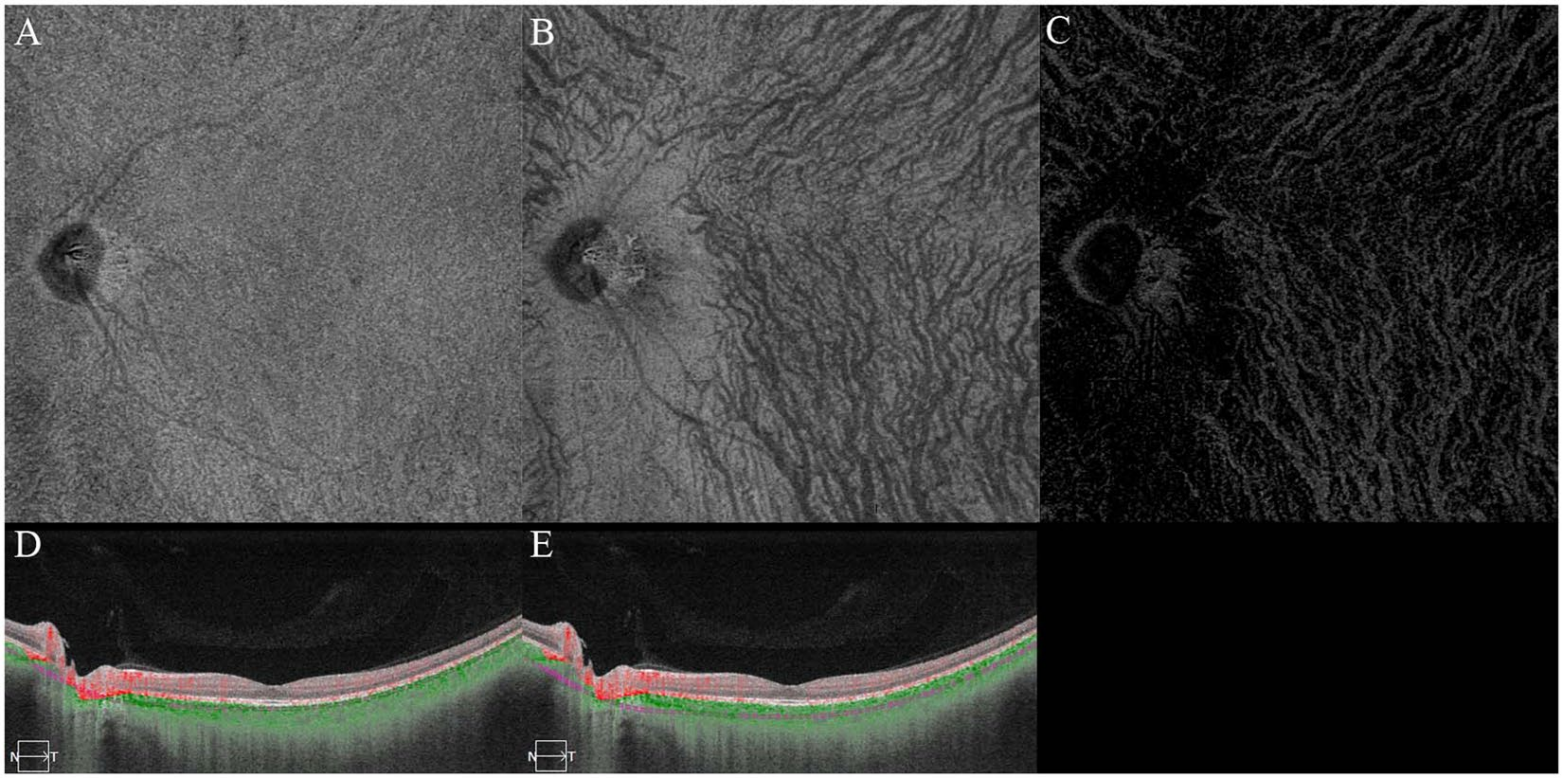
Case 5. Left eye of 30-year-old man. Subfoveal choroidal thickness is 290 µm. Ratio of flow area is 27.4%. Brightness and contrast are adjusted for easier observations of these figures. (**A**) Choriocapillaris image. (**B**) Half of the choroid image. (**C**) Choroidal blood flow image after removal of the projection artifacts of choriocapillaris. (**D**) Cross sectional image with the segmentation boundaries set to the choriocapillaris. (**E**) Cross sectional image with the segmentation boundaries set to one-half of the choroid.

**Figure 2:**
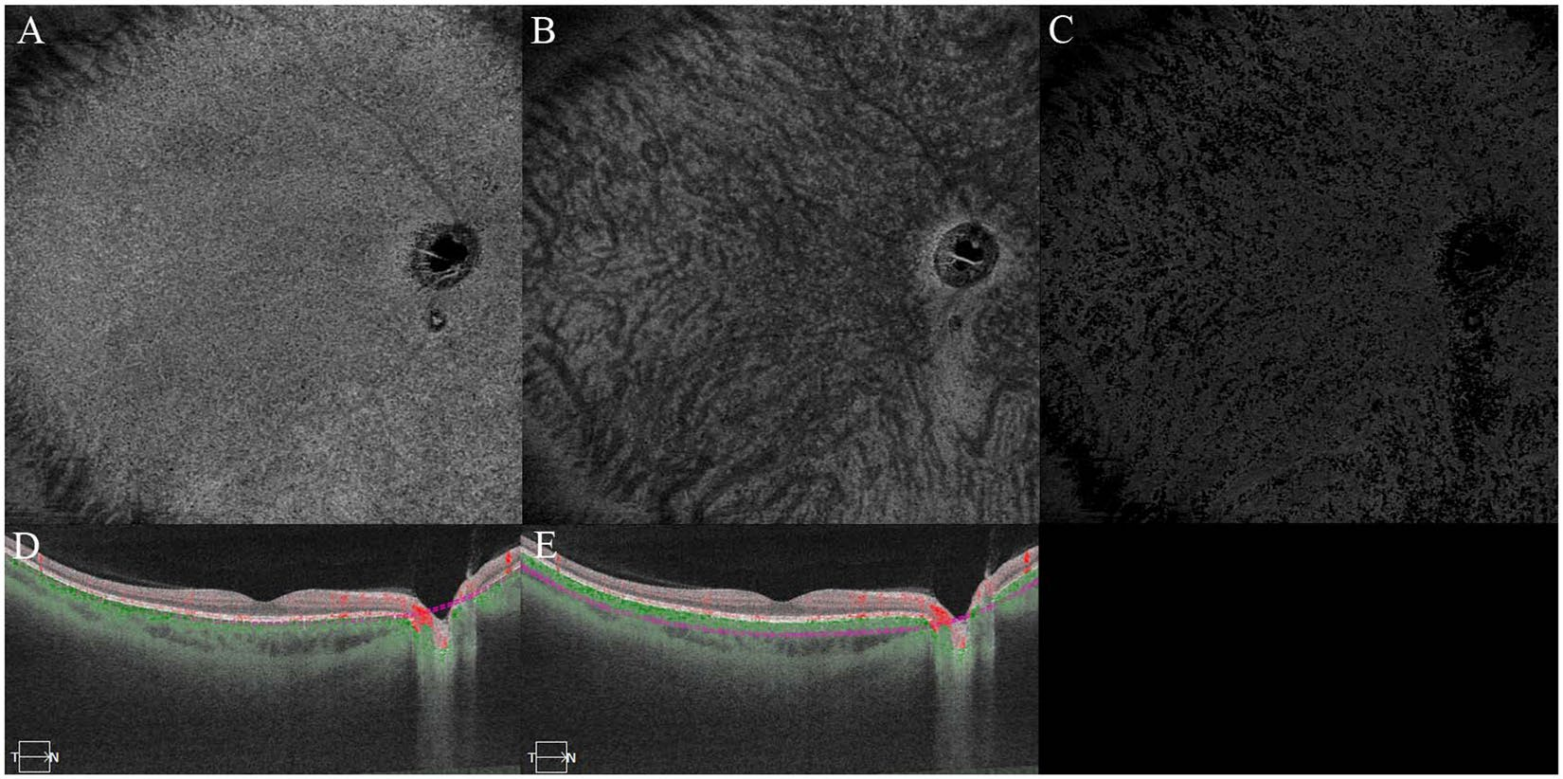
Case 8. Right eye of 43-year-old man. Subfoveal choroidal thickness is 430 µm. Ratio of flow area is 50.1%. Brightness and contrast are adjusted for easier observations of the figures. (**A**) Choriocapillaris image. (**B**) Half of the choroid image. (**C**) Choroidal blood flow image after removal of the projection artifacts of choriocapillaris. (**D**) Cross sectional image with the segmentation boundaries set to the choriocapillaris. (**E**) Cross sectional image with the segmentation boundaries set to one-half of the choroid.

**Figure 3:**
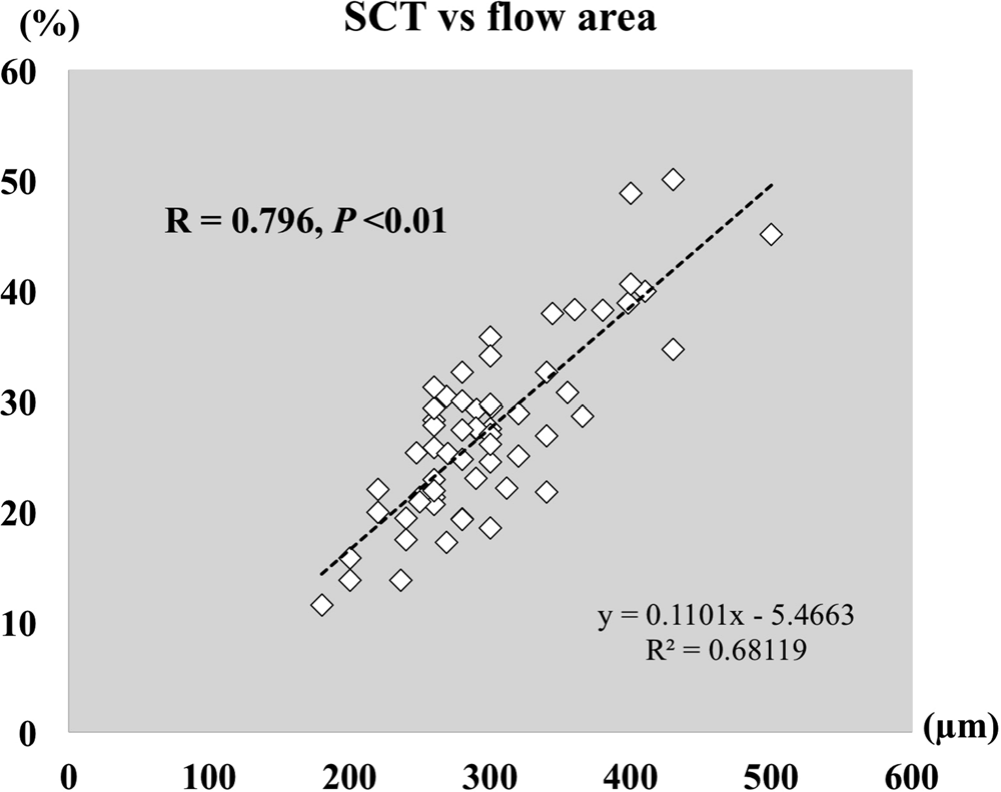
Graph showing the correlation between subfoveal choroidal thickness and ratio of choroidal blood flow. R = 0.796, *P* < 0.01.

**Figure 4:**
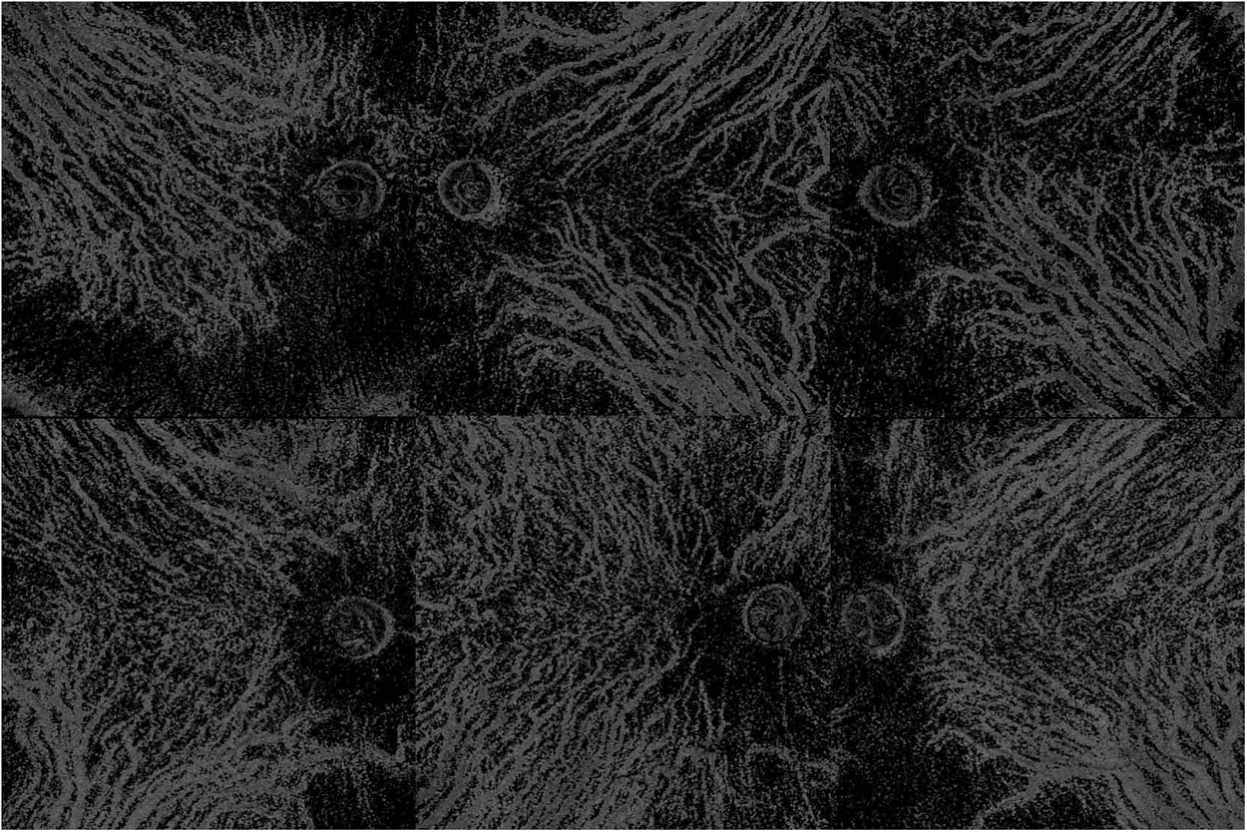
Additional representative choroidal blood flow pattern of the middle and large choroidal vessels. (**A**)Case 13. Right eye. Subfoveal choroidal thickness is 290 µm. Ratio of flow area is 23.0%. (**B**) Case 14. Left eye. 260 µm. 25.8%. (**C**) Case 22. Left eye. 280 µm. 19.3%. (**D**) Case 30. Right eye. 260 µm. 28.3%. (**E**) Case 35. Right eye. 260 µm. 27.8%. (**F**) Case 44. Left eye. 260 µm. 31.2%.

Sixty-one eyes of 45 normal individuals were examined by SS-OCTA. The blood flow pattern, defined as the whitish signal information in the choroidal vessels using OCTA, of the middle and large choroidal vessels was clearly imaged in all 61 eyes in the extracted SS-OCTA images. The mean subfoveal choroidal thickness was 297 ± 61 µm with a range of 180 to 500 µm. The mean ratio of the choroidal blood flow area at the one-half of the choroid (half-choroid) slab (see the detail in the Methods section) was 27.3 ± 8.2% with a range of 11.5 to 50.1%. The findings in all of the eyes are summarized in Table 1, and the data of the left eye of Case 5 are shown in Fig. 1 and that for the right eye of Case 8 in Fig. 2. The ratio of the choroidal blood flow area was strongly correlated with the subfoveal choroidal thickness (R = 0.796, *P* < 0.01; Fig. 3). Additional representative choroidal blood flow pattern of the middle and large choroidal vessels in six eyes are shown in Fig. 4.

**Table 1:** The characteristics of all cases.

Case	eye	OD/OS	gender	age	SCT	flow area %	SD-OCTA
1	1	OD	M	35	290	29.2	+
	2	OS			269	30.4	+
2	3	OD	M	27	247	25.3	+
	4	OS			430	34.7	—
3	5	OD	M	57	355	30.8	—
4	6	OD	M	39	344	37.9	+
	7	OS			398	38.9	+
5	8	OD	M	30	301	29.5	+
	9	OS			290	27.4	+
6	10	OD	F	31	312	22.1	+
7	11	OD	M	67	269	17.2	—
	12	OS			237	13.8	—
8	13	OD	M	43	430	50.1	+
9	14	OD	M	34	366	28.6	+
10	15	OD	F	55	258	21.8	—
11	16	OS	F	30	260	21.3	+
12	17	OD	F	43	180	11.5	+
13	18	OD	F	39	290	23.0	—
14	19	OD	M	38	300	18.5	—
	20	OS			260	25.8	—
15	21	OD	M	42	340	32.6	—
16	22	OD	F	31	380	38.2	—
	23	OS			410	39.9	—
17	24	OS	F	39	260	20.6	+
18	25	OD	F	28	250	21.3	+
	26	OS			250	20.9	+
19	27	OD	F	34	300	27.5	—
	28	OS			340	26.9	—
20	29	OD	F	35	220	22.0	—
	30	OS			220	19.9	—
21	31	OD	F	45	200	15.8	+
22	32	OS	F	45	280	19.3	—
23	33	OD	F	41	270	25.2	+
	34	OS			280	24.7	+
24	35	OR	F	28	290	27.6	—
	36	OS			340	21.7	—
25	37	OS	M	45	280	32.6	+
26	38	OS	F	30	280	19.3	+
27	39	OS	M	32	320	28.9	+
28	40	OS	M	44	200	13.7	+
29	41	OD	F	42	320	25.0	—
30	42	OD	F	38	260	28.3	+
31	43	OS	F	26	280	30.0	—
32	44	OD	F	37	260	22.9	+
	45	OS			300	24.5	+
33	46	OD	F	45	240	19.4	—
34	47	OD	F	43	240	17.4	+
35	48	OD	M	35	260	27.8	—
	49	OS			260	29.3	—
36	50	OD	F	38	300	27.0	—
	51	OS			300	26.1	—
37	52	OD	F	36	280	27.4	+
	53	OS			300	29.7	+
38	54	OD	F	32	500	45.1	+
39	55	OD	M	42	300	35.8	+
40	56	OS	M	38	400	40.6	+
41	57	OD	M	40	300	34.1	—
42	58	OS	F	40	400	48.8	+
43	59	OS	F	46	260	21.9	+
44	60	OS	M	36	260	31.2	+
45	61	OD	M	32	360	38.3	—
Mean ± SD				38.5 ± 8.0	320 ± 62	29.2 ± 8.7	34 eyes
Range				27–67	180–500	11.5–50.1	(37.4 ± 5.6)

SCT (µm) = subfoveal choroidal thickness.

flow area (%) = ratio of choroidal blood flow area by SS-OCTA.

SD-OCTA = spectral domain optical coherence tomography angiography.

**Figure 5:**
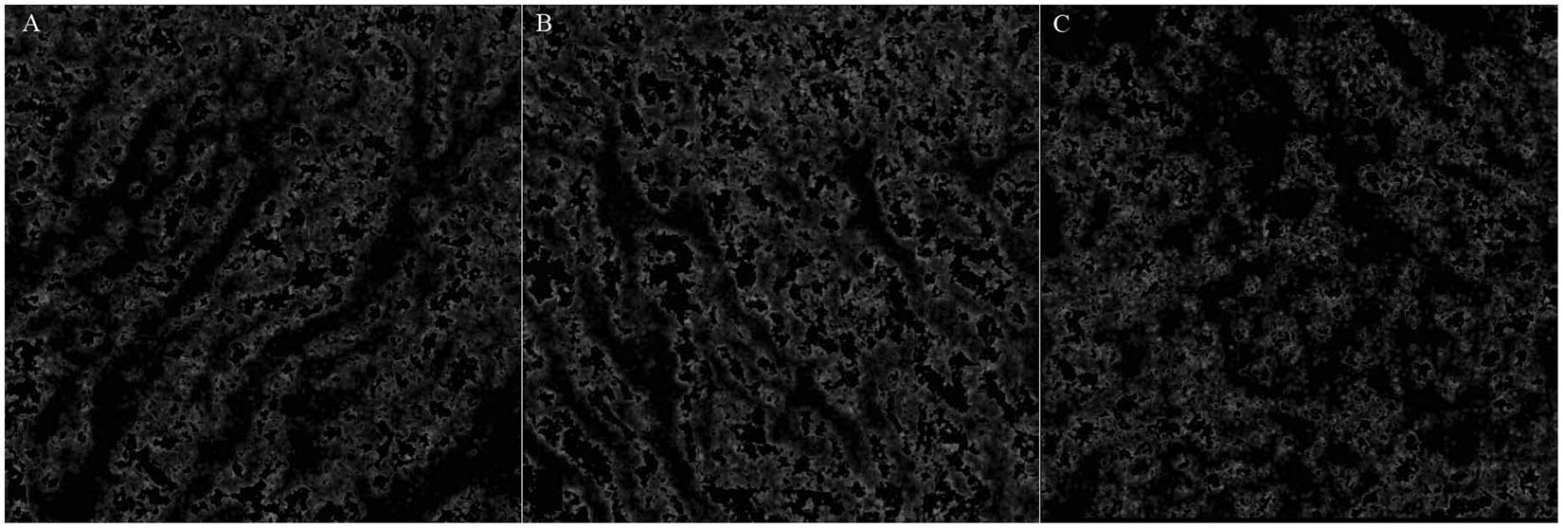
Choroidal blood flow image after removal of the projection artifacts of choriocapillaris using spectral domain optical coherence tomography angiography. Choroidal vessels are not visible in all eyes. Brightness and contrast are adjusted for easy understanding of these figures. (**A**) Case 1. Right eye of 35-year-old subject presented in Fig. 6. (**B**) Case 5 Left eye of (Fig. 1). (**C**) Case 8 Right eye (Fig. 2).

Thirty-four eyes of 27 cases were also examined by SD-OCTA. In the 3 × 3 mm scanned area, the choroidal vessels were not visible in the 34 eyes and almost all vascular areas had no signals. The images obtained by SD-OCTA and extracted in the same way from the representative Case 5 (OS) and Case 8 (OD) are shown in Fig. 5. The ratio of the choroidal blood flow area could not be calculated.

## Discussion

The results showed that the choroidal blood flow pattern could be clearly imaged by SS-OCTA after a segmentation of the half-choroid and removing the projection artifacts of the choriocapillaris. Although imaging the blood flow pattern of the middle and large choroidal blood vessels has been considered to be not possible by the commercially available OCTA instruments, images of the choroidal blood vessel pattern could be recorded at atrophic sites such as the eyes with choroideremia^7^ and/or RPE atrophy^5,8^. This was believed to be possible because the measuring light of the OCT device was able to reach the choroid without attenuation by the RPE. It was also shown that the choroidal blood flow pattern could be obtained without RPE atrophy in eyes with high myopia^6^. Thus, the choroidal blood flow could be imaged by the OCTA instruments if the light of the OCT instrument could reach the choroid. This also indicated that the choroidal blood flow pattern can be imaged even if the flow velocity is slow or turbulent as previously believed^9^.

The choroidal blood flow in the SS-OCTA images was seen as black vessels as if there was no blood flow in the choroidal vessels, and as white in the stromal area. However, choroidal blood flow within the choroidal vessels might be weakly present in the SS-OCTA images. We believed that some blood flow information was present in the luminal area of the choroid if the whitening of the stroma area could be excluded. Thus, we believe the uniformly whitened stromal area of the choroid originates from the choriocapillaris projection artifacts. That is why we developed a method to exclude choriocapillaris projection artifacts. Our results showed that the three subtractions removed the choriocapillaris projection artifacts, and we succeeded in unveiling the blood flow pattern of the middle and large choroidal blood vessels. However, even this method was not successful with the SD-OCTA images most likely because there were no signals in the choroidal blood vessels. This may indicate that our method will not be effective when the OCT signal does not reach deep into the choroid. Our method might not be effective for hypermetropic eyes with a thickened choroid.

This method was simple, however there are limitations as described in the Methods section. This method might acquire the luminance of the white parts of the stromal area of the choroid which is equivalent to the luminance of choriocapillaris, and the luminal area of the choroid had a strong contrast to the stromal area in the choroid. When the stromal area did not appear white due to a thickened choroid and the choroidal blood flow was already displayed as white under special conditions such as RPE atrophy, our subtraction method may not be effective. Another weakness is that the blood flow signal in the slab of choriocapillaris should be equivalent to the blood flow signal by the projection artifact of the choroidal stromal area in the half-choroid slab. Usually the projection artifact should be darker than the original blood flow signal to be targeted, subtracting it in reverse could be a problem. However, our method is not for the removal of the projection artifact of the choroidal stromal area but rather the removal of the signal itself in the half-choroid slab. So we do not think that it will affect the results.

Although the current study was limited to cases with blood flow information of the entire choroid and without RPE atrophy, it was successful in obtaining the choroidal blood flow pattern in normal eyes from the SS-OCTA images. Because the ratio of the choroidal blood flow area was correlated with the SCT, the choroidal blood flow might be one of the key factors for assessing the choroidal thickness. In the future, it may be necessary for the manufacturers of the SS-OCTA devices to add software that will be able to remove the choriocapillaris projection artifacts. Then, it will be necessary for clinicians to evaluate whether determining the choroidal blood flow pattern will be effective for their patients.

## Materials and Methods

This was a retrospective study conducted according to the tenets of the Declaration of Helsinki. The Institutional Review Board of the Tokyo Women’s Medical University School of Medicine approved the procedures used in this study which included OCT observations of eyes with macular and retinal disorders, observational study of age-related macular degeneration, and similar disorders including eyes with normal retinas. Each patient provided informed consent to undergo the standard ophthalmological examinations including OCTA. Approval was also obtained to examine and use all data obtained for future research.

### Choroidal blood flow examinations with swept source optical coherence tomography angiography (SS-OCTA)

Sixty-one eyes of 45 cases (19 men, 26 women; average age, 38.5 ± 8.0 years) without any retinal disorders were examined by SS-OCTA (AngioPlex Elite 9000, Zeiss, Germany) that had a light source with emittance between 1040 nm and 1060 nm. The AngioPlex Elite 9000 has a scan rate of 100,000 A-scans/second with real-time tracking of eye movements for motion-artifact compensation (FastTrac™). These properties greatly improved the clarity of the 12 × 12-mm OCTA *en face* images. The signal detections were based on an optical microangiography (OMAG) algorithm^9^ to evaluate both the signal phase and amplitude changes. The segmentation boundaries and widths can be manually adjusted to select the choroid by the embedded software. The device can also obtain structural OCT scans which can be viewed as B-scan sections or as *en face* slabs corresponding to the thickness used to obtain the OCTA images.

### Segmentation adjustments

The segmentation boundaries were set to the choriocapillaris and half-choroid with a width of 30 µm for the analysis of the *en face* OCTA images. The area of the choriocapillaris was selected to be the region between 30 µm to 60 µm below the outer RPE surface to avoid the projection artifacts of the retinal capillaries. Although the choriocapillaris images are created according to the default-setting from 29 microns to 49 microns (20-micron width) under RPE in Elite 9000, we decided to increase the thickness slightly and calculated it at 30 microns so as not to miss the choriocapillaris signals because the range of the choriocapillaris might vary in each eye. The subfoveal choroidal thickness was determined with the caliper tool in the OCT software. We confirmed not to include the eyes with subfoveal choroidal thinning less than 150 microns at least so as not to enroll the pathologic myopia eyes.

### Subtraction of choriocapillaris projection artifact

**Figure 6:**
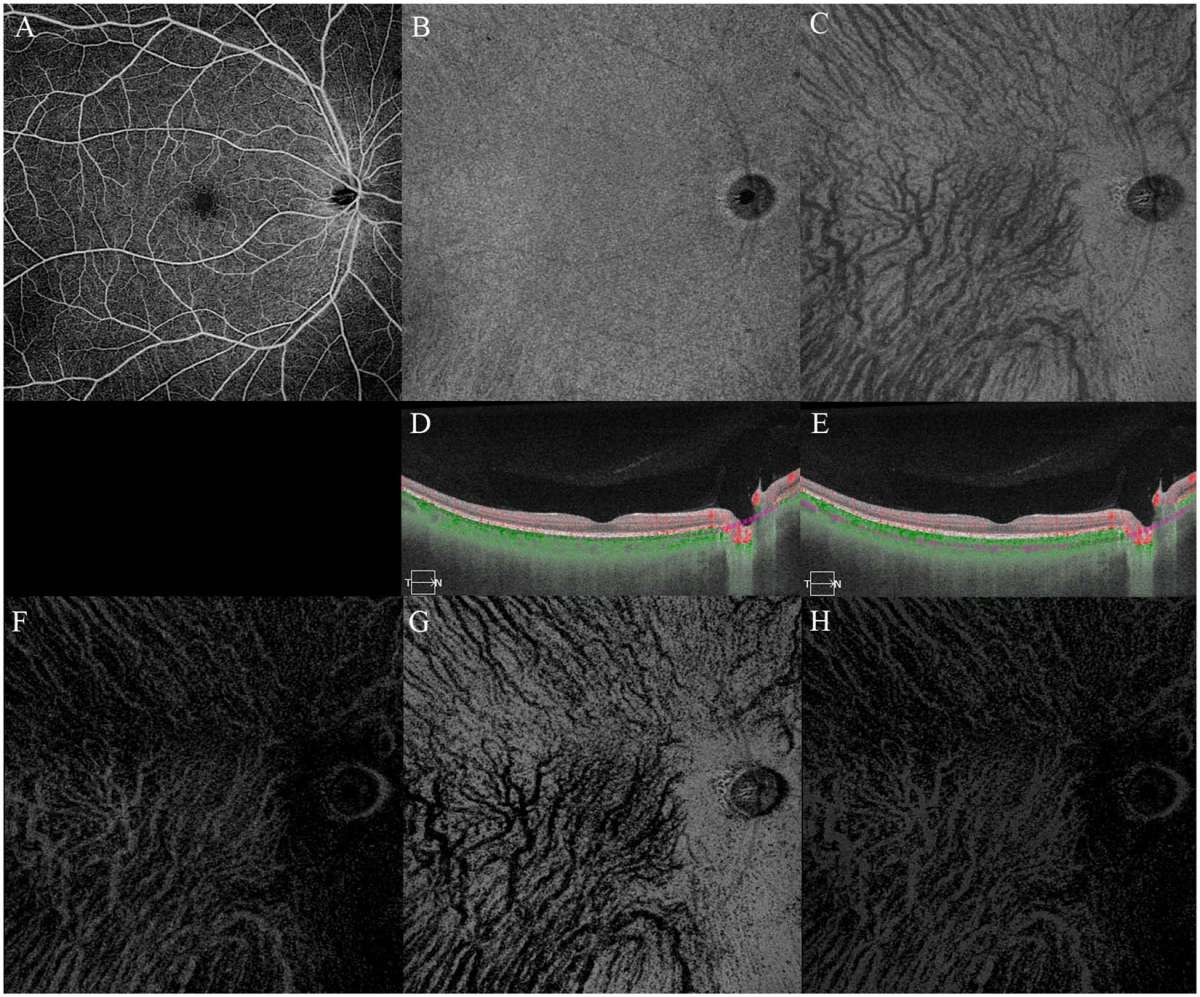
Subtraction of choriocapillary projection artifact. Case 1 was a 35-year-old man. Right eye. Brightness and contrast are adjusted for easier observations of these figures. (**A**) Swept source optical coherence tomography angiographic image of the entire retina. (**B**) Choriocapillaris image. (**C**) Half of the choroid image. (**D**) Cross sectional image with the segmentation boundaries set to the choriocapillaris. (**E**) Cross sectional image with the segmentation boundaries set to one-half of the choroid. (**F**) First subtracted image from the choriocapillaris by C. (**G**) Second subtracted image from the half-choroid by F. (**H**) Third subtracted image from half-choroid by E. Subfoveal choroidal thickness is 290 µm. Ratio of flow area is 29.2%.

After setting the segmentation boundaries in the normal eyes (Fig. 6A), the slab images of the choriocapillaris were made visible (Fig. 6B). Then three subtractions were made to obtain the large choroidal vessel pattern (Fig. 6C). First, the half-choroid image was subtracted from the choriocapillaris image using the ImageJ software developed by Wayne Rasband (National Institutes of Health, Bethesda, MD; available at http://rsb.info.nih.gov/ij/index.html). The first subtracted image was called sub-A (Fig. 6D) which was seen as white choroidal vessels. However, sub-A was not the actual image for choroidal blood vessels. For the second step, sub-A was subtracted from the half-choroid image. The second subtracted image was called sub-B (Fig. 6E) which was a view of the choroidal vessels that is darker than that in the original half-choroid image. The signals at the luminal area in the sub-B were almost zero. Finally, the sub-B image was subtracted from the half-choroid image. The third subtracted image (Fig. 6F) displayed the choroidal blood flow signals in the half-choroid image with the choriocapillaris projection artifacts subtracted out.

A limitation of this technique was that the stromal area in the one-half-choroid was assumed to be the choriocapillaris projection artifact. It was also necessary to assume that the luminance of the one-half choroidal stromal area was approximately the same as the luminance of the choriocapillaris alone. In addition, the contrast between the stromal area and the luminal area in the half-choroid should be twice or more of the difference. These assumptions were reasonable.

### Ratio of choroidal blood flow area

The final image was the choroidal blood flow pattern converted from the RGB image to an 8-bit grayscale image using ImageJ. The ratio (%) of the choroidal blood flow area in the whole choroidal region was calculated after binarization, and the exclusion of the signals around the optic disc. The optic disc in each eye was selected by the oval tool in ImageJ. The binarization was performed by the Bernsen methods of auto local threshold tool on ImageJ. The ratio of the choroidal blood flow area was analyzed to determine whether it was correlated with the choroidal thickness. Spearman’s correlation test was used to determine the significance of the ratio of choroidal blood flow area and subfoveal choroidal thickness.

### Determination of choroidal blood flow by spectral domain optical coherence tomography angiography (SD-OCTA)

Among the 61 eyes, 34 eyes of 27 cases (12 men, 15 women; average age, 37.4 ± 5.6 years) without any retinal disorders were also examined by spectral domain OCTA (SD-OCTA; RTVue XR Avanti, Optovue, Fremont, CA). This SD-OCTA system had an 840 nm light source that could obtain 70,000 A-scans/second with effective motion correction technology. With this system, clear 3 × 3 mm *en face* OCTA images could be obtained. The same segmentation and subtraction of the choriocapillaris projection artifact were performed on these eyes. If the choroidal blood flow pattern can be detected in the modified SD-OCTA images, the ratio of choroidal blood flow area was calculated after the same binarization method. Because the images obtained by SD-OCTA would be within 3 × 3 mm, it was not necessary to eliminate the optic disc.

### Statistical analyses

All *P*-values were two-sided and *P* values < 0.05 were considered statically significant. All statistical analyses were performed with free software EZR (Saitama Medical Center, Jichi Medical University, Saitama, Japan), which is a graphical user interface for R (The R Foundation for Statistical Computing, Vienna, Austria)^10^. More exactly, it is a modified version of R commander designed to add statistical functions frequently used in biostatistics.
